# The Impact of COVID-19 Pandemic on Obsession and Compulsion Symptoms in Saudi Arabia

**DOI:** 10.7759/cureus.20021

**Published:** 2021-11-29

**Authors:** Naseem Alhujaili, Abdulaziz Alghamdi, Tariq Abo Talib, Muhammad Alhaqbani, Mohammad Alfelali, Waleed Alghamdi

**Affiliations:** 1 Department of Medicine, Division of Psychiatry, Faculty of Medicine in Rabigh, King Abdulaziz University, Jeddah, SAU; 2 Department of Medicine, Division of Psychiatry, Faculty of Medicine in Rabigh, King Abdulaziz University, Jeddah 22252, Saudi Arabia., Jeddah, SAU; 3 Department of Family and Community Medicine, Faculty of Medicine in Rabigh, King Abdulaziz University, Jeddah, SAU; 4 Department of Psychiatry, King Abdulaziz University, Jeddah, SAU

**Keywords:** saudi arabia, ocd, obsession and compulsion symptoms, covid-19 pandemic, obsessive compulsive disorder

## Abstract

Background

Obsessive-compulsive disorder (OCD) is a common condition that has a significant impact on people’s lives. COVID-19 pandemic imposed a challenging situation for the general population with new precautionary measures. All that can have serious implications for those who already have intense concerns about cleanliness and hygiene and those diagnosed with OCD. The aim of this study was to examine the impact of the coronavirus disease 2019 (COVID-19) pandemic on the emergence and severity of obsession and compulsion symptoms in Saudi Arabia.

Methods

This was a cross-sectional study with 1,190 participants who completed an online three-part questionnaire that included sociodemographic data, the Yale-Brown Obsessive-Compulsive Scale (Y-BOCS) checklist, and (Y-BOCS) severity scale. On account of the fact that OCD requires a clinical evaluation to confirm the diagnosis, screening positive for OCD was defined based on criteria A of the Diagnostic and Statistical Manual of Mental Disorders (DSM-5), which is selecting at least one of either obsession or compulsion symptoms or both. Screening positive for obsession was defined as selecting at least one of the obsessional symptoms while screening positive for compulsion was defined as selecting at least one of the compulsion symptoms.

Results

Overall, OCD screening was positive in 82% of participants. Previous diagnosis of OCD was reported by 2.6% (N=36) of the participants and 55.2% of them reported that their symptoms did not change during the pandemic, while 41.1% reported that their symptoms increased. Positive OCD screening was significantly higher in participants who reported previous psychological illness (87.6% vs. 80.9%), those who followed news related to COVID-19 on a daily basis (88.7% vs. 76.1%), and participants who had not acquired the infection (82.9%) compared to those who were infected with COVID-19 (72.3%).

Conclusion

The aim of this study was to determine the impact of the pandemic on OCD screening and symptoms. New OCD symptoms were reported in a high proportion of the participants. The results of this study can provide guidance for psychiatrists and psychologists in the clinical approach and management of patients with OCD. Further focused research on the factors affecting the emergence or severity of OCD symptoms needs to be conducted in the future.

## Introduction

At present, coronavirus continues to spread in nations across the world affecting millions of people, without any indication of how or when the disease will come to an end. COVID-19 is a new strain of the coronavirus infection that mainly affects the respiratory system similar to the previous six types, among which were the well-known SARS-CoV and MERS-CoV [1]. COVID-19 was first reported to the world on December 31, 2019, by the World Health Organization (WHO) [2]. By March 2020, the WHO had announced that this disease was a pandemic [3]. By November 1, 2021, the total number of confirmed COVID-19 cases worldwide reached 246,357,468, with 548,571 confirmed cases in Saudi Arabia [4].

Since the onset of COVID-19 in late 2019, a considerable number of studies have focused on the impact of the COVID-19 pandemic on mental health. Several studies have suggested that the COVID-19 pandemic has significant implications for mental health, including anxiety disorders, post-traumatic stress disorder, behavioral disorders, and depression [5-8]. A range of psychiatric manifestations, including psychomotor excitement, psychotic symptoms, delirium, and suicidality have also been reported [9]. An extensive review of the existing literature shows that unpredictability, uncertainty, the seriousness of the disease, misinformation, and social isolation contribute to stress and mental disorders [10,11]. A few studies, including two from Canada, have discussed the mental health impact of the pandemic from the perspective of health anxiety. They found that when people are presented with inaccurate or misleading information during the spread of an infectious disease, it results in maladaptive behaviors such as repeated medical consultations and avoiding healthcare even if genuinely ill. It can even lead to mistrust in public authorities and reduced compliance with instructions [12]. The rates of suicide also went up including an increase in physician suicide as noted by Laboe, C. W. et al. [13].

Obsessive-compulsive disorder (OCD) is a common condition that has a significant impact on people’s lives. According to the Diagnostic and Statistical Manual of Mental Disorders (DSM-5), obsessions are defined as “recurrent and persistent thoughts, urges, or images that are experienced as intrusive and unwanted,” whereas compulsions are defined as “repetitive behaviors or mental acts that an individual feels driven to perform in response to an obsession or according to rules that must be applied rigidly.” Globally, 1.1-1.8% of people suffer from obsessive-compulsive disorder. In the United States, the 12-month prevalence of OCD is 1.2% [14]. However, data from the Saudi National Mental Health Survey showed a higher prevalence of OCD in Saudi Arabia (about 4.1% of the population) compared to the reported prevalence worldwide [15]. This makes it imperative to study the impact of the COVID-19 pandemic on obsession and compulsion symptoms in the Saudi population.

The content of obsessions and compulsions varies between individuals, but common obsessional themes include contamination, symmetry, excessive doubt, or intrusive thoughts that are aggressive, sexual, or intend to harm oneself or others. Common compulsions are repetitive cleaning, ordering, counting, and repetitive checking [14]. Fear of contamination is of interest to this study as it has been reported to be the most common theme in OCD [16]. Additionally, fear of catching the virus and the need to take precautionary measures during the COVID-19 pandemic might specifically heighten contamination-related obsessions and compulsions.

The guidelines from ministries of health affirm that the way to protect oneself from the virus is adherence to cleanliness and social distancing. This can have serious implications for those who already have intense concerns about cleanliness and hygiene and those diagnosed with OCD. The literature showed that fear of COVID-19 has a significantly impacts OCD patients; one indicator can be seen in hoarding; patients start displaying such maladaptive behavior due to fear of unavailability or shortage in the supply of protective measures and equipment. It can also lead to the development of OCD, particularly contamination fear, which can arise from hand hygiene recommendations that are an important protective measure against infection. Although OCD responds well to psychological and pharmacological treatments, the worsening of OCD symptoms may occur during stressful times, particularly under the influence of the frequent reminders emphasizing the importance of cleanliness and hygiene by various sources [17]. It is not surprising that patients with OCD diagnosis doubt the effectiveness of exposure and response prevention therapy (ERP), the first-line psychotherapy for OCD [18,19]. With everyone being asked to frequently wash hands as a precaution against the disease, the pandemic confirms the original fear of contamination that OCD patients have [18]. In a study conducted in Italy on 30 patients diagnosed with OCD before the pandemic, the Yale-Brown Obsessive-Compulsive Scale (Y-BOCS) before and after the quarantine was measured. The Y-BOCS severity score showed a remarkable worsening of OCD symptoms during the quarantine period compared to the pre-quarantine period [20].

A study from Saudi Arabia found that the emergence of new-onset obsessions and compulsion symptoms was higher among employees, housewives, students, and quarantine disciplines. Interestingly, it has been found that those not employed in healthcare had increased obsession compared to medical field workers, while stress levels were higher among those who work in healthcare [21]. Another study from Saudi Arabia concluded that the risk of severe depression during the COVID-19 pandemic was higher among females, single, and young individuals with lower educational levels. In addition, more than half of the participants had unexplained OCD symptoms [22].

We hypothesize that people with or without OCD diagnosis would have experienced the worsening or emergence of obsession and compulsion symptoms during the current COVID-19 pandemic. The main aim of this study was to determine the impact of the COVID-19 pandemic on obsession and compulsion symptoms in Saudi Arabia. Additionally, we examined the association between OCD symptoms during the COVID-19 pandemic with participants' previous diagnosis of psychiatric or chronic medical illness and the magnitude of the COVID-19 news follow-up.

## Materials and methods

This was a cross-sectional study targeting adults aged > 18 years living in Saudi Arabia. An online questionnaire was used and a link to it was posted on social networking sites (Facebook, Twitter, etc.) and cross-platform messaging applications (WhatsApp). The link to the questionnaire was active for two months, from September to November 2020. Informed consent was obtained at the beginning of the questionnaire, which indicated the purpose of the study and the right of the participant to withdraw at any time. The questionnaire had three parts: sociodemographic data, the Yale-Brown Obsessive-Compulsive Scale (Y-BOCS) checklist, and (Y-BOCS) severity scale. 

We collected general sociodemographic data such as gender, age, nationality, city of residence, education, and marital status. In addition to the presence of physical or mental health conditions, being infected with or having contact with a person infected with COVID-19. 

Participants were also asked how frequently they watched news related to COVID-19. In this section, we added a question for those who were diagnosed with OCD before the pandemic asking them to report their perception of the severity of their OCD symptoms before and during the pandemic. The second part of the questionnaire included the Y-BOCS symptoms checklist. We only used the symptoms related to contamination obsessions and cleaning/washing compulsions for simplicity and relevance. Screening positive for obsession was defined as selecting at least one of the obsessional symptoms, while screening positive for compulsion was defined as selecting at least one of the compulsion symptoms. Screening positive for OCD was defined as selecting at least one of either obsession or compulsion symptoms. The questionnaire was translated into Arabic from English and then verified. The internal consistency assessed by Cronbach’s alpha coefficient was α =.85 for obsessional symptoms and α =.78 for compulsion symptoms. The overall alpha coefficient was α =.90 which indicates excellent reliability. In addition, the validity results showed good construct validity [23].

The third part of the questionnaire was the Arabic version of the Y-BOCS, which is a 10-item scale designed to measure the severity of obsession and compulsion symptoms over the past seven days. This scale was validated in Arabic, and the internal consistency assessed by Cronbach’s alpha coefficient was α =.73, indicating good reliability. In addition, the validity results showed good construct validity [23].

The analysis was performed using the Statistical Package for the Social Sciences (IBM SPSS® 25, Armonk, USA). Categorical variables were presented as percentages. Comparisons between groups were performed using the chi-square (x^2^) test and continuous data by Student’s t-test, and appropriate multivariate analysis was performed using multinomial logistic regression to assess demographic and socioeconomic influences on obsession and compulsion symptoms. Statistical significance was set at p ≤0.05. Ethical approval for this study was obtained from the Research Ethics Committee (Reference No 476-20).

## Results

A total of 1,383 participants were assessed for eligibility. Among them, 1,190 participants met the inclusion criteria and agreed to participate in the study. The majority of participants were Saudis (1,137; 95.5%), 796 (66.9%) were female, and 582 (48.9 %) were young adults aged between 18 and 30 years. Detailed characteristics of the participants, including their employment status, educational level, and distribution across regions of Saudi Arabia, are listed in Table 1.

**Table 1:** Characteristics of participants included in the study

Characteristics	Values	Frequencies (%)
Age	18-30	582 (48.9)
	31-40	263 (22.1)
	41-50	151 (12.7)
	51-60	141 (11.8)
	Over 60	53 (4.5)
Gender	Male	394 (33.1)
	Female	796 (66.9)
Nationalities	Saudi	1,137 (95.5)
	Non-Saudi	53 (4.5)
Marital status	Single	514 (43.2)
	Married	605 (50.8)
	Divorced	49 (4.1)
	Widowed	22 (1.8)
Education	None	8 (0.7)
	Primary	2 (0.2)
	Middle	28 (2.4)
	High school	214 (18.0)
	Diploma	94 (7.9)
	College	654 (55.0)
	Higher Education	190 (16.0)
Work status	None	243 (20.4)
	Student	369 (31.0)
	Health professional	156 (13.1)
	Other	313 (26.3)
	Retired	109 (9.2)
Region	Centre	155 (13.0)
	West	735 (61.8)
	East	122 (10.3)
	South	93 (7.8)
	North	85 (7.1)
Infected with COVID-19		101 (8.5)
Had contact with COVID-19 patient		429 (36.1)
Have chronic illness		232 (19.5)
Following news and updates about COVID-19	None	234 (19.7)
	Once a week	351 (29.5)
	More than once a week	270 (22.7)
	Daily basis	335 (28.2)
Were diagnosed with a psychological illness		232 (19.5)

The proportion of participants who were previously diagnosed with any psychological illness was 19.5%. Depression was the most prevalent illness (7.9%), followed by anxiety (5.6%) (Table 2). 

**Table 2:** Frequency and proportion of previously diagnosed psychological illness

Psychological illness	Frequencies (%)
Depression	94 (7.9)
Anxiety	67 (5.6)
Personality Disorders	43 (3.6)
Eating Disorder	37 (3.1)
Obsessive-Compulsive Disorder	36 (3.0)
Psychosis	30 (2.5)
Bipolar	29 (2.4)
Post Traumatic Stress Disorder	27 (2.3)
Other	69 (5.8)

Among those with known cases of OCD (N=36), more than half (55.2%) declared that the symptoms did not change during the pandemic, and 41.1% admitted that the symptoms of OCD had increased during the pandemic.

Strikingly, OCD screening was found to be positive in 82% of the participants. In particular, 78% screened positive for obsession and 43.9% screened positive for compulsion. Positive screening was significantly higher among those who were following news and updates about COVID-19 on a daily basis (88.7%) than among those who did not follow the news (76.1%) (P<0.001). Participants who did not acquire the infection had a higher proportion of positive screening for OCD (82.9%) than those who were infected with COVID-19 (72.3%) (P<0.008) (Table 3).

**Table 3:** Subgroup analysis for Obsessive-Compulsive Disorder (OCD) screening

Characteristics	Values	Frequencies (%) Screened Positive for OCD (N=976)	Frequencies (%) Screened Negative for OCD (N=214)	Chi-square (P-value)
Have chronic illness	Yes	196 (84.5)	36 (15.5)	1.188 (0.276)
	No	780 (81.4)	178 (18.6)	
Were diagnosed with a psychological illness	Yes	169 (87.6)	24 (12.4)	4.807 (0.028)
	No	807 (80.9)	190 (19.1)	
Infected with COVID-19	Yes	73 (72.3)	28 (27.7)	7.098 (0.008)
	No	903 (82.9)	186 (17.1)	
Had contact with COVID-19 patient	Yes	345 (80.4)	84 (19.6)	1.160 (0.281)
	No	631 (82.9)	130 (17.1)	
Following news and updates about COVID-19	None	178 (76.1)	56 (23.9)	16.832 (0.001)
	Once a week	280 (79.8)	71 (20.2)	
	More than once a week	221 (81.9)	49 (18.1)	
	Daily basis	297 (88.7)	38 (11.3)	

The severity of OCD was assessed using the Y-BOCS. Due to the small number of participants in each category of the Y-BOCS scale, we merged the severity categories to mild to extreme levels instead of mild, moderate, severe, and extreme. Those who had been previously diagnosed with a psychological illness had a higher proportion of mild to extreme symptoms of OCD than those who were not diagnosed in the past (42.5% vs. 18.8%, respectively) (P<0.001). 

## Discussion

The present study aimed to determine the impact of the COVID-19 pandemic on obsession and compulsion symptoms in Saudi Arabia. The most important clinically relevant finding was a high prevalence of contamination obsessions and/or cleaning compulsions symptoms during the pandemic (78% and 43.9%, respectively). This high proportion was observed among all the participants, keeping in mind that only 3% of the participants reported having a diagnosis of OCD prior to the COVID-19 pandemic. 

This finding is consistent with other studies conducted in Saudi Arabia, which reported a high prevalence of new-onset obsessions and compulsions during the pandemic [21,22]. A study from Canada reported a 10-to 30-fold increase in OCD symptoms during the pandemic compared to the pre-pandemic prevalence [24]. Multiple studies have examined the relationship between exacerbation of OCD symptoms and the COVID-19 pandemic [21-24]. Fear, uncertainty, unpredictability, and increased stress induced by the pandemic are expected to be the reasons behind the exacerbated OCD symptoms seen in this study. It is possible that the increased level of stress related to the COVID-19 pandemic increased the overall severity of OCD symptoms. The possible mechanism responsible for exacerbating OCD symptoms is excessive checking of news related to COVID-19 that results in increased checking behaviors [24]. Another possible explanation is the extent of anxiety, depression, and experiential avoidance, which have been found to be positively correlated with the increased severity of OCD symptoms. Additionally, fear of being infected by COVID-19 could be directly related to the worsening of OCD symptoms and emotional reactivity. Previous studies have also suggested that fear of getting infected by COVID-19 was indirectly related to the worsening of OCD symptoms possibly because it predicted the worsening of anxiety/depressive symptoms [25]. In our study, depression and anxiety were the most common disorders reported by participants (7.9% and 5.6% of previously diagnosed psychological illnesses, respectively). This supports the notion that increased levels of anxiety and depressive symptoms predict the worsening of OCD symptoms.

It is interesting to note that 55% of the participants with a previous diagnosis of OCD reported no change in the severity of symptoms during the pandemic. Moreover, the Y-BOCS scores showed that 77.4% of the participants with or without OCD were in the subclinical category of the scale, and 22.6 % showed mild to extreme OCD severity. This finding was surprising; higher OCD severity in general and worsening of OCD symptoms among those diagnosed with OCD was expected; however, the results in our study showed the contrary. Even though current studies have mixed results regarding the extent of impact the COVID-19 pandemic has on OCD symptoms, in a recent study, patients with OCD diagnosis showed greater resilience to the worsening of OCD symptoms during the pandemic [26]. In another study, it was found that the level of restrictions related to COVID-19 imposed on the population could play a role in determining the severity or worsening of OCD symptoms during the pandemic. In a prospective cohort, three surveys of OCD symptom severity were administered to participants at three different intervals, during and after the quarantine, four weeks apart. There was a statistically significant correlation between the increased severity of Y-BOCS scores and fear of COVID-19 as reflected by the imposed level of quarantine [27]. The majority of participants in our study scored in the subclinical category on the Y-BOCS scale, possibly because our study was conducted two months after quarantine measures were relaxed and the number of new COVID-19 cases dropped significantly. The majority of the participants reported no previous OCD diagnosis, possibly not having a previous OCD diagnosis could be a protective factor against a more severe form of OCD. However, no previous studies have shed light on this relationship.

The median age of onset of OCD is between 17-22 years. We predicted that the age group between 18-30 would screen positive for OCD more than other age groups [28]. However, no significant difference in the emergence or diagnosis of OCD symptoms was found among the different age groups. In spite of the general notion that OCD is more prevalent in the younger population, this study showed that OCD symptoms were present in similar proportions among all age groups. The COVID-19 pandemic and the crises associated with it affected the entire population; its impact on the emergence of OCD symptoms is still unknown, and recent literature has shown mixed results and the age range is no exception. 

Although the results were not statistically significant, it was surprising that healthcare professionals were the least likely to report OCD symptoms during the pandemic compared to unemployed and non-healthcare employees. Healthcare professionals may benefit from their medical knowledge about the transmission of COVID-19, in addition to the nature of their work that causes repetitive exposure to other infectious agents during their careers. This finding was also reported by Alateeq et al. [21]. This is possibly due to the fact that health care workers have greater knowledge about the disease and its behavior, which suggests that having a higher level of uncertainty and anxiety about the disease increases the chance of reporting or experiencing the worsening of OCD symptoms. 

Surprisingly, no significant correlation was found between chronic medical conditions and OCD positivity. Given the nature of the disease and its ability to affect patients with chronic illness more severely, we expected to find a difference in OCD symptoms between those with chronic illness and those without. We found that the majority of participants who reported having a mental illness diagnosis prior to the pandemic were screened positive for OCD. This also accords with other studies conducted during the pandemic that reported high levels of stress, anxiety, depression, and other illnesses during the COVID-19 pandemic among people with a previous history of mental illness [29].

Another important finding was that the more the participants followed COVID-19-related news, the more likely they were to screen positive for OCD. This finding is consistent with the case report of a 30-year-old female, who reported that the main reason behind her relapse into severe OCD symptoms during the pandemic was increased media reporting of COVID-19 news [30]. Those who followed COVID-19 news on a daily basis and screened positive were 88.8% vs. 11.3% of those who screened negative with a statistical significance of (P=0.001). This finding is in line with the notion that following COVID-19 news is associated with an increased risk of emergence or worsening of OCD symptoms. This can be useful to clinicians when they treat patients with OCD during a pandemic.

The generalizability of these results is subject to certain limitations. For instance, this was a cross-sectional study that indicated the presence of OCD symptoms at a particular time. Therefore, it was beyond the scope of this study to find a causal relationship between the emergence of OCD and the COVID-19 pandemic. Another limitation is that although we used a valid questionnaire for screening and measuring the severity of OCD, the diagnosis of OCD requires a clinical evaluation to confirm the diagnosis. In addition, the questionnaire was self-reported, which carries the risk of recall bias. The generalizability of the results is also limited by the recruitment method, as the questionnaire was distributed through online platforms, so only people who had access to social media were able to participate. 

Further research should be undertaken to investigate the long-term effects of the COVID-19 pandemic on obsession and compulsion symptoms, in addition to comparing the severity level of OCD pre-, during, and post-pandemic. 

## Conclusions

The main goal of the current study was to determine the effect of the COVID-19 pandemic on obsession and compulsion symptoms in Saudi Arabia. The most significant finding was the high proportion of participants (82%) who reported new OCD symptoms; positive screening was higher for obsessions than compulsions. The findings also showed that positive screening was significantly higher among those who were following news related to COVID-19 on a daily basis, had no prior COVID-19 infection, and reported a previous psychiatric diagnosis. An implication of these findings is that they could enable healthcare professionals is to recognize those at higher risk of developing or worsening obsession and compulsion symptoms during the current pandemic and other health-related crises.
